# Sleep Measurement in Osteoarthritis and Inflammatory Arthritis: A Systematic Scoping Review Protocol

**DOI:** 10.1002/msc.70140

**Published:** 2025-06-10

**Authors:** Annalisa De Lucia, Yeliz Prior, Richard Jones, Gianluca Bertoni, Andrea Dell’Isola, Valeria Donisi, Cinzia Perlini, Simone Battista

**Affiliations:** ^1^ School of Health and Society Centre for Human Movement and Rehabilitation University of Salford Salford UK; ^2^ Section of Clinical Psychology Department of Neurosciences Biomedicine and Movement Sciences University of Verona Verona Italy; ^3^ Department of Neurosciences, Rehabilitation, Ophthalmology, Genetics, Maternal and Child Health University of Genoa Campus of Savona Italy; ^4^ Department of Clinical Sciences Lund Clinical Epidemiology Unit, Orthopaedics, Lund University Lund Sweden

## Abstract

**Background:**

Osteoarthritis (OA) and inflammatory arthritis (IA), including rheumatoid arthritis (RA), psoriatic arthritis (PsA), and axial spondylarthritis (axSpA), are leading causes of disability worldwide, significantly impacting health and quality of life. Sleep issues are highly prevalent in these populations, exacerbating pain, fatigue, and disease activity. However, there is a dearth of evidence regarding how sleep disorders should be assessed.

**Objective:**

The main objectives are to identify, describe, and synthesise which types of sleep dimensions are evaluated, what measurement tools are employed to measure them in individuals with OA and IA, and provide an overview of the impact of sleep issues in OA and IA.

**Methods:**

This systematic scoping review will follow the Joanna Briggs Institute methodological framework and be reported using the Preferred Reporting Items for Systematic Reviews and Meta‐Analysis extension for Systematic Scoping Reviews. The search strategy will involve PubMed, Embase, Cochrane Central, PsycINFO, and CINAHL, along with grey literature sources. Articles will be selected based on predefined eligibility criteria, and data will be synthesised narratively.

**Anticipated outcomes:**

This systematic scoping review will synthesise the current literature on studies that collect and report sleep assessment as a primary or secondary outcome in people with OA and IA. It will clarify which sleep dimensions are assessed and how they are measured, provide an updated overview to inform clinical practice regarding sleep assessment and impact in OA and IA, and identify key research gaps.

**Dissemination:**

The findings will be disseminated through research publications, including peer‐reviewed articles and conference abstract(s)/presentation(s).

## 1 Introduction

### 1.1 Burden of Sleep in Arthritis

Arthritis is one of the first common causes of disability worldwide, resulting in a substantial burden on healthcare systems and society (Ackerman et al. 2025; Elgaddal et al. 2022). Among the most frequent and disabling forms of arthritis are osteoarthritis (OA) and inflammatory arthritis (IA), including rheumatoid arthritis (RA), psoriatic arthritis (PsA), and axial spondylarthritis (axSpA) (Black et al. 2023; Steinmetz et al. 2023). These conditions share common clinical features (e.g., pain, fatigue, and reduced range of motion) (Geenen et al. 2018) that often lead to more prevalent sleep issues than in the general population (De Baets et al. 2023; Grant et al. 2023; Salari et al. 2023; Yaseen et al. 2024).

About 70% of people with arthritis report experiencing sleep complaints (Boeselt et al. 2019), such as difficulty in initiating or maintaining sleep, non‐restorative sleep, and daytime sleepiness, often associated with a reduced quality of life and a higher psychological distress, fatigue, pain, and disease activity (Abdelrahman et al. 2024; Gouda et al. 2023; Tighe et al. 2020; Yaseen et al. 2024). Sleep alterations increase the induction of pro‐inflammatory cytokines (Bjurström et al. 2017), potentially exacerbating inflammation processes associated with arthritis and contributing to increased pain (Purabdollah et al. 2017). High levels of pain can then impact sleep quality, leading to a vicious self‐feeding cycle with a worsening of the overall burden of the disease (Boeselt et al. 2019).

### 1.2 Sleep in Arthritis Care: An Underexplored Dimension

Sleep is rarely mentioned in internationally recognised clinical practice guidelines for OA and IA (Bannuru et al. 2019; Moseng et al. 2024; Ramiro et al. 2023; Smolen et al. 2023), even if this topic has become a key area of research in recent years (Feda et al. 2023; Polak et al. 2025).

A systematic scoping review by Feda et al. examined how much sleep is measured in knee OA clinical trials assessing treatment outcomes, showcasing a paucity of sleep data in randomised controlled trials for knee OA (Feda et al. 2023). It is paramount to integrate a comprehensive assessment of sleep quality into the management and treatment strategies of people with OA and IA (Chu et al. 2020; The Lancet Rheumatology 2022) since sleep is a complex construct that can be evaluated across various dimensions (Buysse 2014; Wallace et al. 2018), upon which the development of the RU‐SATED sleep health scale is grounded. This tool allows for the assessment of six distinct dimensions of sleep (i.e., Regularity, Satisfaction, Alertness, Timing, Efficiency, Duration), which are closely linked to significant health outcomes (e.g., mortality, coronary heart disease, hypertension, and depression) (Buysse 2014).

### 1.3 Broadening Perspectives on Sleep in OA and IA: Assessing Multiple Dimensions

To build on and extend the existing evidence, we aim to perform a systematic scoping review that explores the use of validated measurements to assess sleep and related dimensions in OA and IA, regardless of joint location, and considering all study designs and providing an overview of the impact of sleep issues in OA and IA. The explored sleep dimensions retrieved in these studies will be conceptualised in Buysse's framework (2014). This approach will help clarify which sleep dimensions have been investigated, provide an updated state‐of‐the‐art overview to inform clinical practice on sleep measurement and its significance, and identify research gaps that need to be addressed. This work will have important clinical implications by providing evidence to support clinicians in integrating sleep assessments in OA and IA, leading to more personalised and holistic care plans and influencing future clinical practice guidelines for managing these diseases.

### 1.4 Rationale for Conducting a Systematic Scoping Review

While interest in sleep in OA and IA is growing, research in this field remains highly heterogeneous. This is partly due to the variability in both sleep assessment methods, which range from subjective (e.g., questionnaires) to objective measures (e.g., actigraphy and polysomnography), and the specific sleep dimensions examined (e.g., duration, satisfaction, efficiency, etc.). This methodological and conceptual diversity underscores the need for a systematic and comprehensive scoping review to synthesise existing approaches and provide greater clarity in this area of research.

### 1.5 Aim and Objectives

This systematic scoping review aims to systematically map the current literature about studies that collect and assess sleep dimensions as a primary or secondary outcome in people with OA and IA (i.e., RA, PsA, and axSpA) using validated measurements and provide an overview of the impact of sleep issues in OA and IA. It will be framed in a multidimensional sleep health perspective, focussing on the six dimensions defined by the Regularity, Satisfaction, Alertness, Timing, Efficiency, and Duration (RU‐SATED) scale (Buysse 2014; Shuster et al. 2024; Wallace et al. 2018). Specific objectives include:

1. Identifying, describing, and synthesising which sleep dimensions (as defined by the RU‐SATED scale) are evaluated;
2. Exploring how these sleep dimensions are assessed (e.g., patient‐reported outcome measures [PROMs], objective measures, etc.), identifying each assessment method's strengths and limitations, and mapping the frequency of sleep measurement across different study types (e.g., interventional vs. observational studies) and clinical contexts.
3. Providing an overview of the impact of sleep issues in OA and IA.

## 2 Material and Methods

This systematic scoping review will follow the Joanna Briggs Institute (JBI) methodological guidance for systematic scoping review (Peters, Godfrey, et al. 2020; Peters, Marnie, et al. 2020). Reporting will adhere to the Preferred Reporting Items for Systematic Reviews and Meta‐Analyses extension for Systematic Scoping Reviews (PRISMA‐ScR) guidelines and checklist (Tricco et al. 2018).

### 2.1 Research Team

The research team includes healthcare professionals (e.g., clinical psychologists, physiotherapists, epidemiologists, and an occupational therapist) and experts in qualitative and quantitative research, evidence synthesis, rheumatic and musculoskeletal diseases (RDMs), and sleep.

### 2.2 Eligibility Criteria

Eligibility criteria were defined according to the Population, Concept, and Context (PCC) framework proposed by the JBI (Table 1) (Peters, Godfrey, et al. 2020).

**TABLE 1:** Eligibility criteria according to the Population, Concept, Context (PCC) framework.

Population:
Inclusion:
Adults (≥ 18 years old) with a diagnosis of OA and/or IA (i.e., RA, PsA, and axSpA) either clinical (e.g., following specific criteria such as European alliance of associations for rheumatology, EULAR or the National institute for health and care excellence) or instrumental The study must focus primarily on OA and/or IA as the main condition of interest In studies involving more rheumatic and musculoskeletal conditions, we will include samples where it is possible to identify specific subgroups with OA and/or IA
Exclusion:
People younger than 18 years old Absence of a diagnosis of OA and/or IA either clinical (e.g., following specific criteria such as EULAR and ACR) or instrumental Studies in which OA and/or IA are not the primary diagnoses considered Studies where it is not possible to identify subgroups with OA and/or IA
Concept:
Inclusion:
All types of articles reporting primary studies, both quantitative and qualitative (apart from case reports and case series), and protocols with no restrictions on time, geographical location, setting, and language Studies with sleep as a primary or secondary outcome Studies with sleep measured with validated outcome measures (objective or subjective), that is any tool with evidence of validation. For subjective measures (e.g., the Pittsburgh Sleep quality Index (Buysse et al. 1989), the Patient‐reported outcomes measurement information system sleep disturbance (Buysse et al. 2010), the RU‐SATED), validation refers to published psychometric properties (e.g., reliability, validity) in the context of sleep assessment. For objective measures (e.g., actigraphy), validation will require evidence from studies comparing the tool against the gold standard of sleep assessment, polysomnography
Exclusion:
Reviews, editorials, commentaries, expert opinions, letters to editors, case reports, case series, book review chapters, and conference abstracts Studies with sleep that is not a primary or secondary outcome Studies with sleep measured with no validated outcome measures (e.g., single‐item, ad hoc, or non‐validated questions on sleep, qualitative studies)—Unless used in combination with a validated tool
Context:
No specific restrictions on setting

### 2.3 Searching Strategy and Information Sources

Relevant articles will be searched in PubMed, Embase, Cochrane Central, PsycINFO, and CINAHL. The choice was made following the Cochrane Handbook for Systematic Review of Interventions (Higgins et al. 2019), which recommends PubMed, Embase, and Cochrane Central as the bare minimum requirement for conducting a search strategy with other specific databases based on the research question. Considering the topic (Sleep), PsycINFO and CINAHL are both relevant. The search strategy was initially created for PubMed and adjusted to apply to the other databases using Polyglot (Clark et al. 2020). The search strategies were assessed by a Librarian at the University of Salford (See Supplementary Information for the search strategies). A grey literature search will also be carried out using the Canadian Agency for Drugs and Technologies in Health (CADTH) tool for accessing health‐related databases (Canada's Drug Agency 2024). In addition, Clinical.Trial.Gov will be searched to identify any ongoing or upcoming study protocols. Finally, the reference lists of all included studies and relevant reviews will be hand‐searched to identify any further eligible articles (Cross‐reference).

### 2.4 Article Selection

All the retrieved articles will be exported to Covidence (Veritas Health Innovation 2025), where duplicates will be automatically removed. Two blinded reviewers independently assess titles and abstracts for eligibility, followed by the full‐text review. A pilot test, pre‐formal screening for a random 10% of records retrieved, will be conducted as a calibration exercise to improve reliability across reviewers. Formal screening will start if the percentage interrater agreement is > 90%. Otherwise, the inclusion and exclusion criteria will be further specified, and another pilot test will be performed. In case of conflict, a third author will be consulted. The reasons for exclusion will be documented in the systematic scoping review report. The next step will be to systematically map the articles selected as eligible. The study identification and selection process will be represented graphically through the PRISMA flow diagram (Page et al. 2021).

### 2.5 Data Extraction and Synthesis

Data will be extracted from the selected articles by two independent researchers and charted based on an adapted JBI Standardised Data Extraction Form (Peters, Godfrey, et al. 2020; Peters, Marnie, et al. 2020). Then, the two authors will compare the two extraction forms with a third author to determine discrepancies and reach an agreement to a final version. Data extraction will be an iterative process based on the retrieved results. However, we expect to include the following data:

- Authors & year.
- Country of origin.
- Aims/purpose.
- Population and sample size, including clinical characteristics (diagnosis and comorbidities)
- Study design.
- Intervention type, comparator, and details of these (e.g., duration of the intervention, if applicable).
- Sleep dimension(s): Which sleep dimensions have been assessed? The sleep dimensions will be categorised according to the six dimensions reported in the RU‐SATED (Table 2). In addition to these dimensions, sleep architecture will also be considered, including sleep stages (e.g., REM sleep, NREM sleep, and its subdivisions: N1, N2, and N3).
- Methods of sleep assessment: Which type of tools was adopted and what are their psychometric properties (validity, reliability), if reported?
- Key findings from the articles.

The extracted data will be narratively synthesised following the guidance of Popay et al. (Popay et al. 2006).

**TABLE 2:** RU‐SATED health sleep dimensions.

RU‐SATED health sleep dimensions	Explanation
Regularity	Consistent sleep schedule
Satisfaction	Perceived sleep satisfaction
Alertness/Sleepiness	Ability to stay awake during the day
Timing	Sleep occurring at the right time of day
Efficiency	Time spent asleep versus time in bed, latency, wake after sleep onset
Duration	Time asleep

### 2.6 Methodological Quality Appraisal

No critical appraisal of the risk of bias will be conducted in line with the systematic scoping review guidelines (Peters, Godfrey, et al. 2020; Peters, Marnie, et al. 2020).

## 3 Discussion

This systematic scoping review will explore and systematically map the current evidence about studies that collect and report sleep assessment as a primary or secondary outcome in people with OA and IA (i.e., RA, PsA, and AxSpa). The main objectives are to identify, describe, and synthesise which types of sleep dimensions are evaluated, what measurement tools are employed, and provide an overview of the impact of sleep issues in OA and IA. In pursuing these objectives, a multidimensional framework on sleep health will be adopted, focussing on the six sleep dimensions defined by the RU‐SATED scale (Buysse 2014; Shuster et al. 2024; Wallace et al. 2018). This framework allows for a comprehensive examination of sleep across various dimensions, providing a robust foundation for evaluating sleep in these clinical populations.

This protocol follows the established JBI methodological framework for systematic scoping reviews (Peters, Godfrey, et al. 2020; Peters, Marnie, et al. 2020; Tricco et al. 2018). Outlining a clear, step‐by‐step process ensures a systematic and transparent review approach. Any necessary deviations from the protocol will be documented in the ensuing review. It is acknowledged that, as a systematic scoping review, this study will not include a formal quality appraisal of the included studies. This inherent limitation will be taken into account when interpreting the findings, and the descriptive nature of the synthesis will be made explicit in the presentation of results and conclusions.

Findings from this review will have important implications for both research and clinical practice. By mapping the current landscape of sleep assessment in IA and OA, this work will help identify gaps in the literature, such as underrepresented sleep dimensions or inconsistencies in measurement approaches, thereby informing future research directions. Furthermore, this work may inform the development of core outcome sets for sleep in rheumatology research. Clinically, this review will serve as a comprehensive resource for selecting appropriate assessment tools and determining which sleep dimensions should be considered in evaluating and managing sleep issues in these clinical populations, potentially informing clinical practice guidelines.

## Author Contributions

All authors made substantial contributions to the conception or design of the work or the acquisition, analysis or interpretation of data. All authors drafted the work or revised it critically for important intellectual content. All authors approved the version to be published. All authors agree to be accountable for all aspects of the work in ensuring that questions related to the accuracy or integrity of any part of the work are appropriately investigated and resolved.

## Conflicts of Interest

The authors declare no conflicts of interest.

## Supporting information

Supporting Information S1

## Data Availability

The data that supports the findings of this study are available in the supplementary material of this article.
